# Clinical characteristics of pertussis in infants and risk factors for respiratory support

**DOI:** 10.1080/07853890.2025.2514943

**Published:** 2025-06-17

**Authors:** Cui Liru, Wang Jing, Bi Jing, Zhang Yu, Tian Jian, Jiang Min

**Affiliations:** ^a^ Department of Neonatology, Baoding Hospital of Beijing Children's Hospital, Capital Medical University, Baoding, Hebei Province, China; ^b^ Hebei Key Laboratory of infectious Diseases Pathogenesis and Precise Diagnosis and Treatment, Baoding, Hebei Province, China; ^c^ Department of Infectious Diseases, Baoding Hospital of Beijing Children's Hospital, Capital Medical University, Baoding, Hebei Province, China; ^d^ Scientific Research and Education Department, Baoding Hospital of Beijing Children's Hospital, Capital Medical University, Baoding, Hebei Province, China

**Keywords:** Infants, *Bordetella pertussis*, respiratory support, whooping cough

## Abstract

**Objective:**

This study aims to analyze the clinical characteristics, treatment outcomes, and risk factors associated with respiratory support in infants hospitalized with pertussis.

**Patients and methods:**

We retrospectively analyzed the clinical data of 0–1-year-old pertussis patients admitted to Baoding Hospital of Beijing Children's Hospital affiliated with Capital Medical University from January 2022 to May 2024. SPSS27.0 statistical software was used to analyze the differences in data among different groups of children with pertussis and to summarize their clinical characteristics. A multiple logistic regression model was used to analyze the clinical risk factors of the respiratory support group.

**Results:**

We enrolled 233 hospitalized children with pertussis. Children requiring respiratory support had lower vaccination rates and higher incidences of cyanosis, wheezing, and RSV infection. Logistic regression identified age, cyanosis after coughing, and IVIG use as independent predictors of respiratory support. Age was an independent protective factor: older children were less likely to require respiratory support (OR = 0.151). Compared with children aged ≥3 months, children aged <3 months had a higher history of contact with cough patients, with symptoms such as cyanosis after coughing, white blood cell counts (WBCs) ≥20 × 10^9^/L, lymphocyte percentage ≥60%, and increased RSV infection incidence. Rates of respiratory support, bronchoscopy treatment, IVIG, tracheal intubation, and exchange transfusion treatment increased (all *p* < 0.05).

**Conclusions:**

Younger pertussis patients have more severe clinical manifestations, with significantly increased WBCs, and they are more likely to be infected with other viruses. Age is an independent protective factor, and the younger the patient, the more likely they are to require respiratory support. These findings highlight the need for early recognition and targeted interventions, particularly in younger infants with severe symptoms.

## KEY MESSAGES

- This study analyzed the clinical characteristics, treatment, and outcomes of 233 hospitalized children aged 0–1 years with pertussis, revealing significant differences between those requiring respiratory support and those who did not.
- The findings indicated that children in the respiratory support group had a lower vaccination rate and experienced more severe symptoms, including cyanosis, respiratory distress, and higher incidences of RSV complications.
- Age emerged as an independent protective factor, with younger children being more likely to require respiratory support and experiencing more severe clinical manifestations of pertussis.

## 1. Introduction

Pertussis, or whooping cough, is a highly infectious acute respiratory disease caused by *Bordetella pertussis* (*B. pertussis*). It is characterized by paroxysmal spasmodic cough, coughing violently, a red face, cyanosis of the lips, and, in severe cases, asphyxia. It is commonly complicated by pneumonia, acute respiratory distress syndrome, pulmonary hypertension, or pertussis encephalopathy, increasing the risk to young children [1]. Since the inclusion of the combined diphtheria-tetanus-pertussis vaccine in the Expanded Program on Immunization in 1974, the incidence of pertussis has decreased significantly. However, in recent years, in many countries with high vaccine coverage (including the United States, the European Union, etc.), after vaccination against pertussis, the incidence rate increased again after the decline, which is called 'pertussis reappearance' [2]. Pertussis has reappeared on a large scale worldwide, mainly affecting infants and adolescents. Due to incomplete immunity of infants to pertussis, they have the highest incidence, hospitalization rates, and fatality rates [3]. The World Health Organization estimates that around 160,000 children under the age of five died from Pertussis in 2014 [4]. Pertussis is in the top 10 for disease burden in children under 10 years of age in 2019 [5]. European data show that the incidence of infants <1 year old is 73.6 per 100,000, of which the hospitalization rate of infants <3 months old is as high as 63.1%, and that of infants 3-5 months old is 30.3% to 61.4% [6]. The incidence of pertussis in China from 2018 to 2022 ranged from 0.32 to 2.71 per 100,000, with 52.40% of infants <1 year old [7,8]. Although pertussis has been extensively studied [9–11], the current literature mainly focuses on the epidemiological ­characteristics and the impact of vaccination, the risk factors of respiratory support needs in severely ill children, and its association with the age of the children, immune status, and auxiliary examination. For example, a high white blood cell count (WBC) is listed as a risk factor for severe illness, but its direct correlation with respiratory failure still needs to be verified. In addition, differences in medical resources between regions may lead to differences in the implementation rate and effectiveness of respiratory support. Through retrospective analysis of clinical data of hospitalized children with pertussis aged 0–1 years, this study aims to determine the clinical predictors of respiratory support in infants with pertussis, hypothesizing that younger age, cyanosis, and co-infections contribute to increased severity.

## 2. Methods

### 2.1. Patients

#### 2.1.1. Ethics

The study was approved by the Medical Ethics Committee of the Baoding Hospital of Beijing Children's Hospital, Capital Medical University (approval number 2024-44). This study complied with the *Helsinki Declaration* of 1975, as revised in 2000, and all subjects were exempt from informed consent by the Medical Ethics Committee of the Baoding Hospital of Beijing Children's Hospital, Capital Medical University.

#### 2.1.2. Study design

This was a retrospective study. The medical records of children aged 0–12 months who were admitted to a Children's Hospital with pertussis from January 2022 to May 2024 were retrospectively analyzed.

#### 2.1.3. Inclusion and exclusion criteria

The inclusion criteria were as follows: (1) confirmed diagnosis of pertussis according to the Pertussis Diagnosis and Treatment Protocol (2023 version) [5]; and (2) age 0–12 months.

The exclusion criteria were as follows: (1) unconfirmed cases; (2) presence of severe congenital malformations, genetic diseases, or chromosomal abnormalities; (3) automatically discharged when the condition did not improve; (4) readmitted to the hospital within 2 weeks of discharge; and (5) transferred cases were considered the same case.

#### 2.1.4. Grouping method

Patients were divided into two groups according to whether respiratory support was needed. Patients in the respiratory support group received either invasive support in the form of normal- or high-frequency mechanical ventilation or non-invasive ventilator support in the form of nasal continuous positive airway pressure, bilevel positive airway pressure, or non-invasive positive pressure ventilation. Patients in the non-respiratory support group received either nasal catheter oxygen inhalation or no oxygen inhalation. Patients were also stratified according to age into the <3 months group and the ≥3 months group.

Relevant definitions and diagnostic criteria:

Polymerase Chain Reaction(PCR) technology: Using a segment of DNA as a template, with the joint participation of DNA polymerase and nucleotide substrates, this segment of DNA is expanded to a sufficient quantity for structural and functional analysis. Clinical signs: Refer to the pertussis Diagnosis and Treatment Protocol (2023 Edition) [12].

### 2.2. Technical information

#### 2.2.1. Data collection

Data on patient characteristics, including sex, age, weight, pertussis vaccination status, and possible contacts with infected individuals, and clinical information, including symptoms, laboratory analysis, antibiotic use, respiratory support, tracheoscopy, treatment, hormone use, intravenous immunoglobulin (IVIG) use, length of hospital stay, and hospitalization costs were collected from the electronic medical record system of the Children's Hospital.

#### 2.2.2. Specimen collection

Within 24 h of admission, 1–2 ml of sputum from the lower respiratory tract was collected from all patients through aseptic negative-pressure sputum extraction performed by trained medical personnel. Peripheral venous blood was also collected from all patients within 24 h of admission, and further blood samples were collected as needed. Blood samples were used for routine analysis as well as analyses of blood gases, electrolytes, and biochemistry.

#### 2.2.3. Pathogen detection

Detection of *B. pertussis* nucleic acid: Qualitative detection of pertussis nucleic acid in samples was performed using a real-time (RT) fluorescence quantitative polymerase chain reaction (PCR) instrument (Roche series-LightCycler480, Roche Cobas z 480; Applied Biosystems series-Applied Biosystems 7500). For the experiment, 1 mL of sterile saline was added to the nasopharyngeal swab-containing tube, vortexed and shaken for 15 s. Then, 200 µL of the solution was transferred to an EP tube. DNA extraction followed the QIAamp DNA Mini Kit (QIAGEN) protocol. The PCR primers and probes were synthesized in line with the Monitoring Technical Manual of the National Pathogen Identification Network Laboratory. PCR conditions: 1 cycle at 50 °C for 2 min, 95 °C for 15 min, then 40 cycles of 94 °C for 15 s, 55 °C for 45 s. Positive and negative controls were run with samples for assay validation. During testing, the Ct value (indicating nasopharyngeal bacterial DNA load) was calculated. A Ct < 38 and a plateauing/near-plateauing amplification curve meant a positive result; otherwise, negative.

12 link detection of respiratory pathogens: This test uses the RT fluorescent quantitative PCR method and an automatic medical PCR analysis system to conduct qualitative analysis of the nucleic acids of 12 types of bacteria in the samples. It involves three areas: reagent preparation, sample preparation, and library amplification. For the same batch of samples to be tested, positive and negative quality controls are added, and each well has a corresponding internal control. If the positive quality control shows an amplification curve and Ct ≤ 36, while the negative quality control shows no amplification curve or Ct > 36, the experiment is valid, and subsequent analysis of the sample results can be carried out. Otherwise, re - amplification is required. When a typical S-shaped amplification curve is detected in the detection channel of each sample and Ct ≤ 36, it indicates that the corresponding pathogen is positive. If Ct > 36 or there is no Ct value, it is negative. The bacteria to be detected include Escherichia coli, Staphylococcus aureus, Pseudomonas aeruginosa, Klebsiella pneumoniae, Enterobacter cloacae, Haemophilus influenzae, Streptococcus pneumoniae, Acinetobacter baumannii, Mycobacterium tuberculosis, Pseudomonas maltophilia, Legionella pneumophila, and Methicillin-resistant Staphylococcus aureus.

Respiratory virus 13 link detection: This test utilizes the combined technology of multiplex RT-PCR and capillary electrophoresis. An automatic capillary electrophoresis system is employed to conduct qualitative analysis of the nucleic acids of 13 types of viruses in the samples. It involves four areas: reagent preparation, sample preparation, library amplification, and capillary electrophoresis. Positive and negative quality controls are added to the same batch of samples to be tested. Only after the quality control passes can the results of the peaks be interpreted. Thirteen sets of specific primers are used for one-step RT-PCR amplification in a single amplification tube. Capillary electrophoresis is used to separate the amplification products of different lengths, and the detection results of the pathogens are obtained. By simultaneously detecting human RNA and human DNA in the samples, the entire detection process, including nucleic acid extraction, RT-PCR, and capillary electrophoresis, can be monitored. The 13 respiratory pathogens to be detected include influenza virus subtype H1N1, influenza virus subtype H3N2, coronavirus, chlamydia, respiratory syncytial virus, metapneumovirus, adenovirus, Mycoplasma pneumoniae, rhinovirus, parainfluenza virus, influenza A virus, influenza B virus, and bocavirus.

Blood biochemical test: The operation of Beckmann AU5811 automatic biochemical analyzer uses selective electrode (indirect method) to measure blood sodium, rate method (lactate-pyruvate, L-P) to measure LDH, γ-GT rate method (γ-glutamyl 3 carboxyl 4 nitroaniline method) to measure γ-GT.

## 3. Statistical analysis

Statistical analyses were performed using SPSS software version 27.0. Normally distributed continuous variables are expressed as mean ± standard deviation ( 𝜒� ± s) and were compared using t-tests. Non-normally distributed continuous variables are expressed as median (interquartile range) [M(P25, P75)] and were compared using Mann-Whitney U tests. Count data are expressed as numbers (%) and were compared using chi-squared tests (*n* ≥ 40, *T* ≥ 5), correction formulas (*n* ≥ 40, 1 ≤ *T* < 5), or Fisher's exact tests (*n* < 40 or *T* < 1). *p* < 0.05 was considered statistically significant. Multiple logistic regression analysis was used to identify risk factors for the need for respiratory support in infants with pertussis.

## 4. Results

### 4.1. Patient characteristics

**Figure 1.**
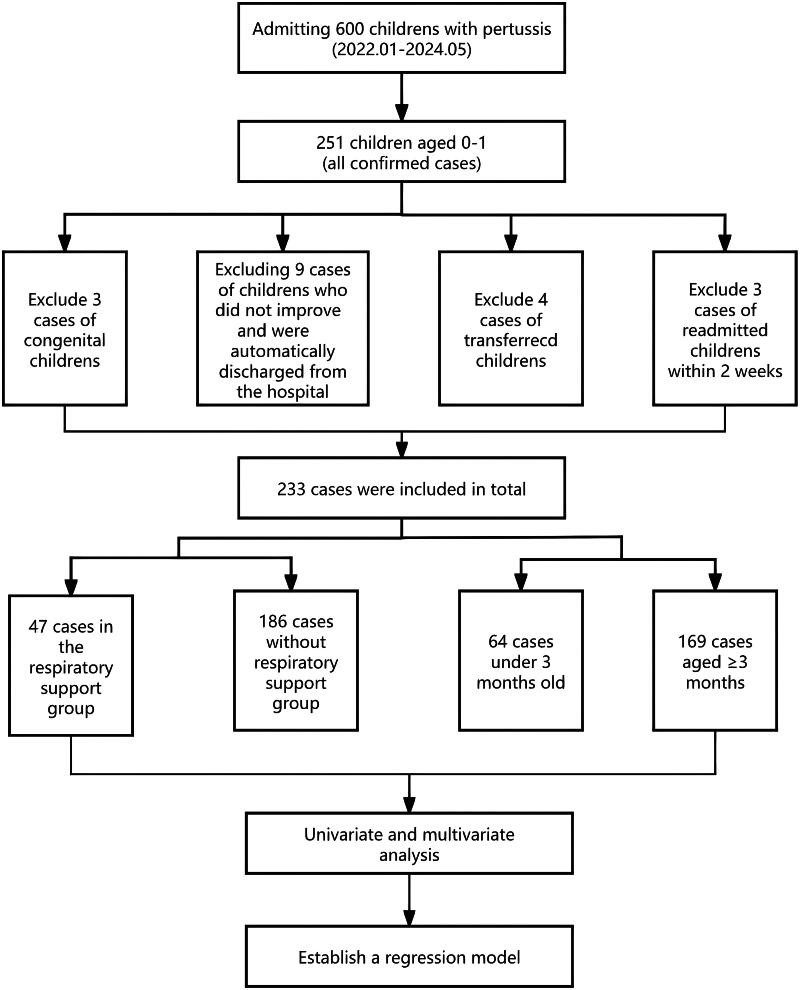
Flow chart of pertussis.

**Figure 2.**
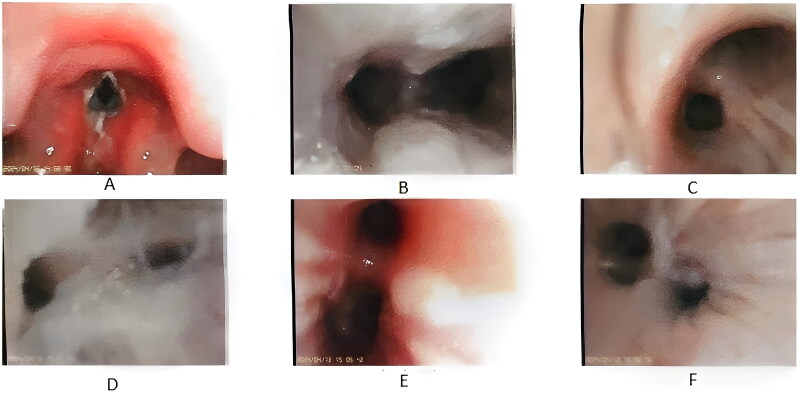
Bronchoscopy manifestations of the deceased child: necrotic epithelium attached to the vocal cords, rough and swollen tracheal mucosa with white secretion attached, and necrotic epithelium attached to the left main bronchus. Note: (a) Glottis, (b) bulge, (c) upper and lower lobes of the right lung, (d) superior lobe of right lung, (e) upper and lower lobes of the left lung, (f) basal segment of inferior lobe of left lung.

**Figure 3.**
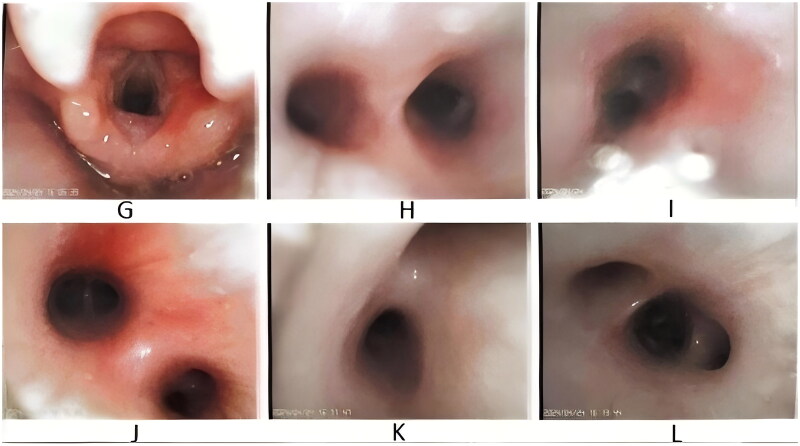
Bronchoscopy manifestations of another surviving child on mechanical ventilation: rough and swollen tracheal mucosa with white discharge attached. Note: (a) Glottis, (b) bulge, (c) upper and lower lobes of the left lung, (d) inferior lobe of left lung, (e) upper and lower lobes of the right lung, (f) middle lobe and lower lobe of right lung.

From January 2022 to May 2024, 573 patients with pertussis were admitted to the Children's Hospital, including 251 children aged 0–1 years. Three children with congenital malformations were excluded, 9 children were excluded for automatic discharge without improvement, 4 children were transferred to other departments as repeated cases, and 2 children were excluded for readmission within 2 weeks (Figure 1). A total of 233 patients with a confirmed diagnosis of pertussis were included in this study, of whom 139 were male and 94 were female. There were 34 children who used antibiotics before inclusion, accounting for 14.6%. All patients had cough, with a mean duration of 14.3 ± 12.2 days at admission. Of the 233 patients, 47 received respiratory support (20.2%), and 186 did not. There were 64 patients (27.5%) aged <3 months and 169 patients (72.5%) aged ≥3 months (Figure 1). In 56 patients (24.0%), pertussis was the only infection present, whereas other infections were also detected in 177 patients (76%). In terms of treatment, 175 patients (75.1%) were treated with erythromycin, 47 (20.2%) with azithromycin, 66 (28.3%) with compound sulfamethoxazole, and 128 (54.9%) with other antibiotics, including second/third-generation cephalosporins, amoxicillin clavulanate potassium and latoxefin sodium. A total of 17 patients (7.3%) received invasive mechanical ventilation, 16 (6.9%) underwent bronchoscopy and alveolar lavage, 16 (6.9%) received IVIG therapy, 11 (4.7%) received methylprednisolone anti-inflammatory therapy, and 3 (1.3%) received blood exchange therapy. One patient (0.43%) died after receiving extracorporeal membrane oxygenation, and the remaining 232 patients were discharged following improvement in their conditions. The median length of hospital stay was 9.34 (6.7, 15.8) days. Figure 2 shows the bronchoscopy manifestations of the patient who died, and Figure 3 shows the bronchoscopy manifestations of a patient who received invasive mechanical ventilation and survived.

### 4.2. Comparison between the respiratory support and non-respiratory support groups

#### 4.2.1. Comparison of clinical characteristics

The age at onset and vaccination rate were significantly lower in the respiratory support group than in the non-respiratory support group (*p* < 0.05 and *p* < 0.01, respectively). The incidence of cyanosis after coughing, fever, wheezing, shortness of breath, laboured breathing, and respiratory failure; the utilization rates of IVIG, hormones, tracheoscopy, and invasive mechanical ventilation; and hospitalization costs and length of hospital stay were significantly higher in the respiratory support group than in the non-respiratory respiratory support group (*p* < 0.05, *p* < 0.01), as shown in Table 1.

**Table 1.** Comparison of clinical characteristics between respiratory support group and no respiratory support group.

Items	Respiratory support group (*n* = 47)	No respiratory support group (*n* = 186)	χ²/Z	*p*
On admission age (month)^b^	2 (1,3)	4 (3,6)	−5.275	<0.001
Gender (male)^a^	29 (62.2)	110 (59.1)	0.143	0.705
Get vaccinated	5 (10.6)	71 (38.2)	12.941	<0.001
A spasmic cough	37 (83.0)	160 (86)	0.417	0.519
Contact history of patients with cough	16 (35.6)	47 (25.3)	1.933	0.164
Flush on face after coughing	40 (88.9)	156 (83.9)	0.71	0.4
cyanotic after coughing	17 (36.2)	9 (4.8)	28.613	<0.001
Fever	18 (38.3)	37(19.9)	5.028	0.025
Wheezing	23 (51.1)	60 (32.3)	5.594	0.018
Shortness of breath	25 (53.2)	16 (8.6)	46.659	<0.001
Dyspnoea	33 (74.5)	12 (6.5)	103.330	<0.001
Apnoea of breath	2 (4.3)	0	4.151	0.195
Respiratory failure	20 (42.6)	0	75.216	<0.001
Positive sputum culture	9 (19.1)	31 (16.7)	0.032	0.858
Erythromycin	33 (72.3)	142 (76.3)	0.179	0.672
Azithromycin	15 (31.9)	32 (17.2)	4.394	0.036
Compound sulfamethoxazole	19 (40.4)	47 (25.3)	2.831	0.092
Combination with other antibiotics	38 (80.9)	90 (48.4)	14.605	<0.001
Bronchoscope treatment	16 (34.0)	0	56.257	<0.001
Use of IVIG	14 (29.8)	2 (1.1)	41.697	<0.001
Use of hormones	10 (21.3)	1 (0.5)	28.606	<0.001
Tracheal intubation	17 (36.2)	0	60.929	<0.001
Hospitalization days (days)^b^	14.2 (9,20.5)	7 (6,10)	−10.005	<0.001
Hospitalization cost (RMB)^b^	26605.39 (15737.63,38540.60)	6302.50 (4989.09,8109.49)	−9.809	<0.001

^a^ Cases (%).

^b^ [M(p25,p75)], [M(p10,p90)].

IVIG: intravenous immunoglobulin.

#### 4.2.2. Comparison of laboratory indicators

Analysis of laboratory indicators revealed that gamma-glutamyl transferase (GGT) levels were significantly higher, and serum sodium levels were significantly lower in the respiratory support group than in the non-respiratory support group (both *p* < 0.05), as shown in Table 2.

**Table 2.** Comparison of laboratory indicators between respiratory support group and no respiratory support group.

Items^a^	Respiratory support group (*n* = 47)	No respiratory support group (*n* = 186)	χ²/Z	*p*
Highest WBC (×109/L)	15.82 (11.025,22.815)	13.19 (9.43,20.327)	−1.953	0.051
Highest L per (%)	9.62 (6.76,15.395)	8.745 (5.92,14.712)	−0.87	0.384
Highest L count (×109/L)	69.2 (62.55,7.11)	72.75 (64.52,79.3)	−1.632	0.103
Highest γ-GT (u/L)	41 (27.5,74.5)	22 (15,31)	−5.399	<0.01
Highest LDH (u/L)	320 (291,397)	308.50 (269.75,364.00)	−1.794	0.073
Lowest Serum sodium (mmol/l)	136 (134.5,138)	138 (137,139)	−4.224	<0.01

^a^ [M(p25,p75)].

WBC: white blood cells; L count: lymphocytes count; L per: lymphocytes percent; γ-GT: gamma-glutamyl transpeptidase; LDH: lactate dehydrogenase.

#### 4.2.3. Comparison of merging with other pathogens

The incidence of respiratory syncytial virus infection was significantly higher in the respiratory support group than in the non-respiratory support group, whereas the incidence of *Streptococcus pneumoniae* infection was significantly lower in the respiratory support group than in the non-respiratory support group (both *p* < 0.05), as shown in Table 3.

**Table 3.** Comparison of combined with other pathogens between respiratory support group and no respiratory support group.

Items^a^	Respiratory support group (*n* = 47)	No respiratory support group (*n* = 186)	χ²/Z	*p*
RSV	11 (24.4)	19 (10.2)	6.492	0.011
RV	12 (26.7)	40 (21.5)	0.553	0.457
HI	6 (13.3)	12 (6.5)	1.526	0.217
SP	3 (6.7)	37 (19.9)	4.427	0.035
MP	1 (2.2)	16 (8.6)	1.329	0.249
AV	1 (2.2)	3 (2.6)	0.079	0.779
IV	1 (2.2)	11 (5.9)	0.393	0.531
PIV	3 (6.7)	14 (7.5)	0.039	0.843
NCV	1 (2.2)	0	4.151	0.195
EV	2 (4.4)	1 (0.5)	4.314	0.098
OP	13 (28.9)	53 (28.5)	0.003	0.958

^a^ Cases (%).

RSV: respiratory syncytial virus; RV: rhinovirus; HI: haemophilus influenzae; Sp: Streptococcus pneumoniae; MP: Mycoplasma pneumoniae; AV: adenovirus; IV: Influenza virus; PIV: Parainfluenza virus; NCV: novel coronavirus; EV: enterovirus; OP: other pathogens.

#### 4.2.4. Multivariate logistic regression model analysis of the impact on the outcomes of children in the respiratory support group and the non-respiratory support group

Multivariate logistic regression model analysis was performed using the variables with statistical significance in the above univariate analyses as independent variables and respiratory support as the dependent variable (no = 0, yes = 1). Age, cyanosis after coughing, fever, shortness of breath, heavy breathing, and hormone and IVIG treatment were identified as independent influencing factors for respiratory support. Age was a protective factor, with older patients being less likely to require respiratory support (odds ratio [OR] = 0.151). Patients with clinical manifestations, such as cyanosis after coughing, fever, shortness of breath, and heavy breathing, were more likely to require respiratory support. The odds ratios were 5.626, 5.655, 15.163, and 16.270, respectively. The probability of requiring respiratory support was 52.204 times higher in patients who received hormones than in those who did not receive hormones and 16.679 times higher in patients who received IVIG than in those who did not receive IVIG. The assignment of each variable and the results of the multifactor analysis are presented in Tables 4 and 5.

**Table 4.** Single factor variable assignment with statistical significance between respiratory support group and no respiratory support group.

Factors	Variable names	Assignment description
Age (month)	x1	<3 = 1, 3-6 = 2, 7-12 = 3
Get vaccinated	x2	No =0, yes =1
Cyanotic after coughing	x3	No =0, yes =1
Fever	x4	No =0, yes =1
Wheezing	x5	No =0, yes =1
Shortness of breath	x6	No =0, yes =1
Dyspnoea	x7	No =0, yes =1
Combination with other antibiotics	x8	No =0, yes =1
Bronchoscope treatment	x9	No =0, yes =1
Use of hormones	x10	No =0, yes =1
Use of IVIG	x11	No =0, yes =1
Co-infection with RSV	x12	No =0, yes =1
Respiratory support	y	No =0, yes =1

RSV: respiratory syncytial virus; IVIG: intravenous immunoglobulin.

**Table 5.** Multivariate logistic regression model analysis affecting respiratory support.

Influencing factors	βi	SE	Wald-*χ*^2^	*p*	OR	95% CI
On admission age (month)	−1.894	0.573	10.904	0.001	0.151	0.049–0.463
Cyanotic after coughing	1.727	0.743	5.409	0.020	5.626	1.312–24.120
Fever	1.733	0.854	4.114	0.043	5.655	1.060–30.166
Shortness of breath	2.719	0.753	13.038	<0.001	15.163	3.466–66.334
Dyspnoea	2.789	0.643	18.801	<0.001	16.270	4.611–57.405
Use of hormones	3.955	1.582	6.249	0.012	52.204	2.349–1160.015
Use of IVIG	2.814	1.401	4.038	0.044	16.679	1.702–259.597

βi: partial regression coefficient; SE: standard error; IVIG: intravenous immunoglobulin.

### 4.3. Comparison between the <3 months and ≥3 months groups

#### 4.3.1. Comparison of clinical characteristics

The incidence of possible contacts with infected individuals, cyanosis after coughing, wheezing, breathing difficulties, respiratory failure, a white blood cell count of ≥20 × 10^9^/L, a lymphocyte percentage of ≥60%, use of IVIG, bronchoscopy, and invasive mechanical ventilation, as well as hospitalization costs and length of hospital stay were significantly higher in the <3 months group than in the ≥3 months group (*p* < 0.05 or *p* < 0.01), as shown in Table 6.

**Table 6.** Comparison of clinical characteristics between <3 months group and ≥3 months group.

Items	<3 months group (*n* = 64)	≥3 months group (*n* = 169)	χ²/Z	*p*
On admission age (month)^b^	1.58 ± 0.53	5.63 ± 2.55		
Gender (male)^a^	33 (51.6)	106 (62.7)	2.327	0.127
Get vaccinated	0	76 (45%)	41.553	<0.001
A spasmic cough	51(79.7)	148(87.6)	2.637	0.104
Contact history of patients with cough	26(40.6)	38(22.5)	7.276	0.007
Flush on face after coughing	52(81.3)	146(86.4)	1.165	0.28
Cyanotic after coughing	16(25.0)	10(5.9)	13.528	<0.001
Fever	13(20.3)	42(24.9)	1.297	0.255
Wheezing	18(28.1)	65(38.5)	1.752	0.186
Shortness of breath	17(26.6)	24(14.2)	3.227	0.072
Dyspnoea	28(43.8)	19(11.2)	27.242	<0.001
Apnoea of breath	2(3.1)	0	2.738	0.268
Respiratory failure	16(25.0)	4(2.4)	25.795	<0.001
Combined with other organ damage	23 (35.9)	42 (24.9)	2.560	0.11
White blood cell count ≥20 × 10^9^/L	45(70.3)	51(30.2)	28.848	<0.001
Lymphocyte percentage ≥60%	57(89.1)	129(76.3)	4.288	0.038
Erythromycin	44 (68.8)	132 (78.1)	1.892	0.169
Azithromycin	19 (29.7)	28 (16.6)	4.419	0.036
Compound sulfamethoxazole	24 (37.5)	42 (24.9)	2.560	0.11
Combination with other antibiotics	43 (67.2)	85 (50.3)	4.586	0.032
Respiratory support	30 (46.9)	17(10.1)	35.631	<0.001
Bronchoscope treatment	12 (18.8)	4 (2.4)	12.769	<0.001
Use of IVIG	9 (14.1)	7 (4.1)	4.382	0.036
Use of hormones	4 (6.3)	7 (4.1)	0.053	0.818
Tracheal intubation	11 (17.2)	6 (3.6)	7.268	0.007
Hospitalization days (days)^b^	9 (6.75,15.25)	8 (6,10)	−2.452	0.014
Hospitalization cost (RMB)^b^	11670.12 (7424.23,8241.00)	6482.77 (5067.95,8868.26)	−5.952	<0.001

^a^ Case (%).

^b^ [M(p25,p75)].

IVIG: intravenous immunoglobulin.

#### 4.3.2. Comparison of laboratory indicators

Analysis of laboratory indicators revealed that γ-GT levels were significantly higher, and serum sodium levels were significantly lower in the <3 months group than in the ≥3 months group (both *p* < 0.05), as shown in Table 7.

**Table 7.** Comparison of laboratory indicators between <3 months group and ≥3 months group.

Items^a^	<3 months group (*n* = 64)	≥3 months group (*n* = 169)	χ²/Z	*p*
Highest WBC (×10^9^/L)	14.20 (9.54,20.00)	13.62 (9.560,21.325)	−0.097	0.923
Highest L per (%)	70.95 (64.75,78.03)	72.10 (62.95,79.15)	−0.167	0.868
Highest L count (×10^9^/L)	9.11 (6.31,14.10)	8.93 (5.91,15.12)	−0.209	0.835
Highest γ-GT (u/L)	50.5 (34,80.75)	19 (14,28)	−9.237	<0.001
Highest LDH (u/L)	316 (281,357)	310 (273.5,367)	−0.079	0.937
Lowest Serum sodium (mmol/l)	137 (135,138)	138 (137,139)	−3.79	<0.001

^a^ [M(p25,p75)].

WBC: white blood cells; L count: lymphocytes count; L per: lymphocytes percent; γ-GT: Gamma-glutamyl transpeptidase; LDH: Lactate dehydrogenase.

#### 4.3.3. Comparison of merging with other pathogens

The incidence of respiratory syncytial virus infection was significantly higher in the <3 months group than in the ≥3 months group (*p* < 0.05), as shown in Table 8.

**Table 8.** Comparison of combined with other pathogens between <3 months group and ≥3 months group.

Items^a^	<3 months group (*n* = 64)	≥3 months group (*n* = 169)	χ²/Z	*p*
SCP	14 (21.9)	26 (15.4)	1.008	0.315
RSV	14 (21.9)	16 (9.5)	6.902	0.009
RV	12 (18.8)	40 (23.7)	0.484	0.487
HI	5 (7.8)	13 (7.7)	0.009	0.925
SP	6 (9.4)	34 (20.1)	3.454	0.063
MP	2 (3.1)	15 (8.9)	1.376	0.241
AV	0	4(2.4)	1.493	0.576
IV	1 (1.6)	11 (6.5)	1.326	0.250
PIV	1(1.6)	16 (9.5)	3.033	0.082
NCV	1(1.6)	0	2.738	0.268
EV	2 (3.2)	1 (0.6)	2.455	0.176
OP	24 (37.5)	44 (26)	1.984	0.195

^a^ Cases (%).

SCP: sputum culture positive; RSV: respiratory syncytial virus; RV: rhinovirus; HI: Haemophilus influenzae; Sp: Streptococcus pneumoniae; MP: Mycoplasma pneumoniae; AV: adenovirus; IV: influenza virus.

## 5. Discussion

The results of this study suggest that patients aged <3 months are more likely to experience severe disease and require respiratory support than those aged ≥3 months. Age, cyanosis after coughing, fever, shortness of breath, heavy breathing, and hormone and IVIG treatment were identified as independent influencing factors for respiratory support, with age serving as a protective factor and clinical signs such as cyanosis as risk factors. However, the two variables of IVIG and hormones are more likely to represent sicker infants were more likely to receive these treatments, rather than the treatments themselves worsening the disease.

In recent years, more and more reports on pertussis have received widespread attention from society [13–16]. The incidence of pertussis has increased due to insufficient persistence of vaccine-induced immunity, differences in vaccination strategies, changes in epidemiological characteristics, antibiotic resistance, pathogenic variation, and improvements in diagnosis and surveillance [17]. In our hospital, 16 positive cases were identified from 429 submitted for testing in 2021, 2177 positive cases were identified from 4888 submitted for testing in 2022, 299 positive cases were identified from 1322 submitted for testing in 2023, and from January to May 2024, 1406 positive cases were identified from 3894 submitted for testing. These figures are consistent with the reported epidemiological characteristics of pertussis in China [18].

Previous studies have found that the transmission route of pertussis gradually changes from adolescents and adults to newborns and infants [19]. In the present study, 63 patients (27.0%) had been in contact with potentially infected individuals. As the family members of the patients had no recent history of pertussis vaccination, we highly suspect that subclinical or inapparent infections were present in these individuals. Therefore, indirect protective measures such as maternal pertussis vaccination are important for reducing pertussis infections in young infants [20]. In this study, 27.5% of patients were aged <3 months. The incidence of exposure history, cyanosis after coughing, wheezing, respiratory distress, respiratory failure, white blood cell count ≥20 × 10^9^/L, and lymphocyte percentage ≥60%, as well as the use of immunoglobulin, bronchoscopy treatment, and invasive mechanical ventilation in the <3 months group were higher than those in the ≥3 months group. The hospitalization costs and duration were higher than those in the ≥3 months group. Children with pertussis, especially those under 3 months of age, had more severe clinical symptoms and were more difficult to treat. These results are consistent with the findings of a foreign single-centre study of 144 children with pertussis, of whom 92% of the children aged <3 months required hospitalization, and 88% developed severe pertussis [21]. Several other studies have also shown that young age is a risk factor for pneumonia or severe pertussis in children with pertussis and that the younger the age, the more likely the disease is to be severe. In this study, the proportion of patients aged <3 months was significantly higher in the respiratory support group than in the non-respiratory support group. Univariate analysis showed statistical significance and multivariate logistic regression revealed that age is an independent protective factor. The older an individual is, the less likely they are to need respiratory support, which is consistent with other scholars' reports [22–24]. The analysis was performed for the following reasons: (1) active immunization without vaccination; (2) immunological immaturity in young children; (3) thoracic airway stenosis and poor cough strength; and (4) pertussis toxin can cause delayed ciliary movement, increased secretion but poor clearance ability, and eventually airway obstruction and even asphyxia [11,25]. Therefore, this research team suggests that the relevant government departments adjust the immunization schedule and advance the vaccination time of the DPT combined vaccine. In this study, the levels of γ-GT were higher in the respiratory support group and the age <3 months group than in the non-respiratory support group and the age ≥3 months group, and the blood sodium levels were lower in the non-respiratory support group and the age ≥3 months group. At present, the specific pathogenesis of pertussis complicated with hyponatremia remains unclear but may be related to the inflammatory response induced by pertussis-related toxins causing capillary leakage, promoting water and sodium entry into the interstitium [26]. In addition, patients aged <3 months receiving respiratory support may experience dyspnoea and loss of appetite, reducing their sodium intake. Severe pertussis may also be accompanied by the abnormal secretion of antidiuretic hormones.

The standard pertussis vaccination schedule in China involves vaccinations at 3, 4, 5, and 18–24 months of age. Studies have shown the effectiveness of three doses and one dose for the successful prevention of pertussis in children aged 6–23 months to be 91.7% and 46.0%, respectively [27]. In this study, only 32.6% of patients were vaccinated, with this figure rising to 89.4% of patients in the respiratory support group in the single factor analysis, significantly higher than the proportion in the non-respiratory support group, suggesting that pertussis vaccination reduced clinical symptoms and the requirement for respiratory support. However, multivariate logistic regression analysis did not identify vaccination as an independent risk factor for respiratory support, in contrast to the results of previous studies [28,29]. Concomitant infection with *B. pertussis* and other pathogens can make treatment more challenging and prolong hospital stays. Therefore, the presence of additional pathogens should be actively investigated. In this study, the pathogens most commonly found alongside *B. pertussis* in the respiratory support group were rhinovirus and respiratory syncytial virus; thus, excessive use of non-macrolide antibiotics should be avoided. In this study, 41.2% of the children had white blood cell counts ≥20 × 10^9^/L, and the incidence of white blood cell counts ≥20 × 10^9^/L was higher in the age <3 months group than in the age ≥3 months group. As pertussis toxin causes leukocytosis by promoting the entry of lymphocytes into the blood, this result indirectly reflects the activity of pertussis toxin [30]. Abnormally increased white blood cell counts, coupled with poor deformation, slow pulmonary blood flow and can block the narrow alveolar capillary bed, causing pulmonary hypertension, hypoxaemia, and even heart failure [31]. Domestic and foreign studies have shown that hyperleukocytosis is a predictor of death in children with severe pertussis [29,32,33]. In this study, the highest white blood cell count, 59.47 × 10^9^/L, was observed in the blood of the patient who died, and even after two blood exchanges, this count remained at 38.79 × 10^9^/L. This patient also experienced acute respiratory distress syndrome and heart failure and died despite receiving extracorporeal membrane oxygenation. Therefore, monitoring the white blood cell count during treatment is of great significance for disease evaluation and guiding rescue treatment.

This study has certain limitations. First, *B. pertussis* cultures and drug sensitivity tests were not performed, so treatment was not targeted. Second, the retrospective design inherently carries risks of selection bias and residual confounding, despite our rigorous statistical adjustments. Third, treatment decisions were individualized per clinician judgement, reflecting real-world heterogeneity but potentially introducing confounding by indication. The last, as a single-centre study, our findings may be influenced by local protocols and patient demographics, necessitating external validation. Future prospective, multicentre studies with protocol-driven treatments are essential to confirm these results.

## 6. Conclusions

In summary, children under 3 months of age with pertussis are the key prevention and control targets. This group has severe clinical symptoms, is prone to other infections, has a long disease course, and is associated with high medical costs. For children with clinical manifestations such as cyanosis, fever, shortness of breath, and difficulty breathing after coughing, as well as those with significantly increased white blood cell counts, effective treatment measures should be actively taken to reduce the occurrence of severe illness and complications. Popularizing pertussis vaccination can help reduce the occurrence of the disease.

## Data Availability

The datasets generated and/or analyzed during the current study are not publicly available due to privacy requirements; however, the data are available from the corresponding author on reasonable request.
